# Comparison of the Hardness of Novel Experimental Vinyl Poly Siloxane (VPS) Impression Materials with Commercially Available Ones

**DOI:** 10.1155/2022/1703869

**Published:** 2022-02-09

**Authors:** Shahab Ud Din, Farooq Ahmad Chaudhary, Bilal Ahmed, Mohammad Khursheed Alam, Sandra Parker, Mangala Patel, Muhammad Qasim Javed

**Affiliations:** ^1^School of Dentistry (SOD), Federal Medical Teaching Institution (FMTI)/PIMS, Shaheed Zulfiqar Ali Bhutto Medical University (SZABMU), Islamabad, Pakistan; ^2^Centre for Oral Bioengineering (Dental Physical Sciences Unit), Bart's and the London School of Medicine and Dentistry, Queen Mary University of London, UK; ^3^Preventive Dentistry Department, College of Dentistry, Jouf University, 72345 Sakaka, Saudi Arabia; ^4^Department of Conservative Dental Sciences, College of Dentistry, Qassim University, Al-Qassim, Saudi Arabia

## Abstract

**Purpose:**

To determine the hardness and Young's moduli of both commercial and experimental vinyl poly siloxane (VPS).

**Methods:**

The purpose of this study was to develop a medium-bodied experimental (Exp-I, II, III, IV, and V) VPS impression materials and to analyse their effects on hardness and Young's modulus and compare them with three commercial VPS materials (Aquasil, Elite, and Extrude) using Shore A hardness tester. Measurements were recorded after 1, 24, 72, and 168 hours of mixing. The results were analysed with one-way ANOVA and post hoc Tukey's test using the SPSS PASW statistical 22 software.

**Results:**

Commercial and experimental vinyl polysiloxane exhibited higher Shore A hardness values with time (i.e., 1 hour after mixing, 24 hours after mixing, 72 hours after mixing, and 1 week after mixing). All Comml and Exp VPS demonstrated a significant increase (ANOVA, *p* < 0.05) in hardness at increasing time points. Generally, all commercial VPS exhibited significantly higher values for Shore A hardness compared to all Exp formulations. For commercial products, Elt M presented significantly highest values at all-time points followed by Aq M then Extr M. Exp-I was significantly harder than all other Exp VPS at all-time points. Young's modulus values were directly related to Shore A hardness; materials with higher Shore A hardness values had higher Young's moduli.

**Conclusion:**

Continued polymerisation of elastomeric impression materials results in increased hardness over time. Hardness, Young's moduli, and rigidity of the set commercial and experimental VPS materials were within the required limits. Shore A hardness and Young's moduli were directly proportional to each other, and commercial and experimental materials had enough rigidity to contain the stone during pouring.

## 1. Introduction

The ability of a material to resist surface indentation or penetration is called its hardness [1]. A variety of tests are available to measure hardness. These tests are defined by the geometry and dimensions of their indenters and the amount of load applied. The load per unit surface area of the indentation gives the hardness number [2, 3]. Brinell, Knoop, Vicker, Rockwell, and Barcol are used to measure the hardness of rigid materials such as metals, alloys, and rigid restorative dental materials. Unfortunately, these methods cannot be used for elastic materials, such as elastomers, where the deformation is elastic rather than permanent [1].

The force necessary to remove the impression from the mouth is directly related to the hardness of the impression material [1]. The hardness also changes with time for some materials (e.g., vinyl polysiloxane: Aquasil). Elastomers are characterised according to their hardness. This is why it is one of the most important and most studied characteristics of these materials. The most commonly used methods are the International Rubber Hardness Degrees (IRHD) and Shore hardness test. A wide range of Shore durometers has been described by ASTM D2240 with Shore A hardness being the most appropriate for the measurement of hardness of elastomeric impression materials. The Shore A hardness scale increases from low to high viscosity [4, 5].

### 1.1. Relation between Hardness and Young's Modulus

The ability of a material to resist deformation under stress due to its stiffness is referred to as its Young's or elastic modulus [6]. Young's modulus of an elastomeric impression material can be calculated from its hardness value. Meththananda et al. evaluated the relationship between hardness and Young's moduli of some elastomeric impression materials where they measured the Shore A hardness of VPS and one polyether impression material [2]. Young's moduli were calculated from the hardness values using the following equations: *H* = 100erf(*kE*^1/2^), where *H* is hardness value, *k* is 3.186 × 10^−4^ Pa^−1/2^, *E* is Young's modulus, and the erf is the error function (to generate a hardness scale), and

(1) $$\begin{array}{c} E\left(\text{MPa}\right)=\frac{0.0981\left(56+7.66\text{s}\right)}{0.137505\left(254-2.54\text{s}\right)}, \end{array}$$

where *s* is Shore A hardness. The direct method is a measure of stress/strain in tension. The first equation gave more accurate values (closer to the direct method). The two equations were compared with each other and with the Young's moduli calculated by the direct method (Table 1) [2, 7].

The elastic modulus of elastomeric impression materials of similar viscosity increases in the following order: polysulphides, condensation silicone, VPS, and polyether [8].

Six commercially available impression materials, three VPS (Aquasil light, Honigum light, President Plus Jet), two condensation silicone (Rapid Liner, Detaseal light), and one polyether (Impregum F), were evaluated by Papadogiannis et al. for Young's modulus [9]. The materials were stored at room temperature (22°C) for 30 min, 60 min, 3 hours, 24 hours, 48 hours, 1 week, and 2 weeks period time. Their results showed that Young's moduli increased for all materials investigated with increasing storage time. The Young's moduli ranged from 1.81 to 12.99 MPa, with the polyether material being the stiffest (10.63-12.56 MPa), followed by VPS and finally condensation silicones.

The objectives of this study were to develop novel experimental VPS impression materials to improve their hardness, other mechanical, and wetting properties. The details about the other mechanical properties such as tear strength, tensile strength, and wetting properties are given in the published part of this study [10–13]. The newly formulated five experimental VPS impression materials containing an additional cross-linking agent (tetra-functional (dimethylsilyl) orthosilicate (TFDMSOS)) and a novel surfactant, Rhodasurf CET-2 (ethoxylated cetyl-oleyl alcohol), were compared with three commercial VPS impression materials. The hypothesis of the current study was that the additional cross-linking agent (TFDMSOS) will form a further bond of crosslinking leading to improvement in the mechanical properties as it happened in the cases of tear strength [11, 13] and tensile strength [10]. The novel surfactant (Rhodasurf CET-2) would also chemically bond to the polymer matrix as a result of its chemical structure and would further improve the hardness of these materials [11]. It is known that the stiffer impression materials (with high Young's modulus) cause difficulty in removing the impression (and tray) from the mouth [2, 14].

It is important to note that strain-in-compression is another sort of hardness test, and the values of strain-in-compression of an impression material should be within the normal range of ISO4823 (2007) [15]. Hence, impression materials should not be very stiff yet they should have enough rigidity to contain the stone during pouring.

## 2. Materials and Methods

Hydrophilic commercially available VPS impression materials were used in this study (Table 2) [16]. To standardize the mixing ratios, they were supplied in auto-mixed cartridges of medium-body consistency.

### 2.1. Preparation of Experimental VPS Impression Materials

Tetra-functional dimethylsilyl orthosilicate (TFDMSOS) and Rhodasurf CET-2 which are cross-linking agents were added to improve tear strength. Nonionic surfactant ethoxylated cetyl-oleyl alcohol was added to improve the wetting. Different compositions were prepared, and out of 113 experimental formulations, only five formulations were suitable for use and were labeled (Exp-I, II, III, IV, and V). The formulations and their details are present in the published part of the present research [16, 17]. For the following formulations, the catalyst paste was the same (Exp-I and II). The control for Exp-II was Exp-I. The Exp-II was used as a control for Exp-III, IV, and V. The formulations for (Exp-III, IV, and V) the catalyst paste were the same.

**Figure 1:**
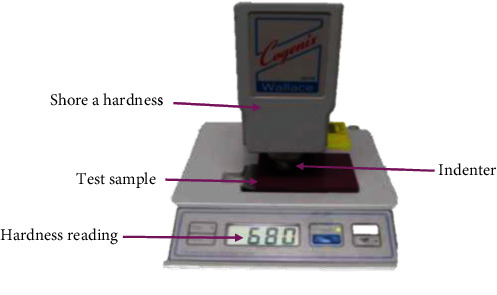
A typical Shore A hardness set up of Aq M in a Shore A.

The hardness of both commercial and experimental was determined by using the Shore A hardness tester (H17A, Congenix Wallace, Kingston, England) in accordance with ASTM:D2240 [17] (Figure 1). Standard rubber test reference blocks supplied by the manufacturers were used to calibrate the equipment before each use.

**Figure 2:**
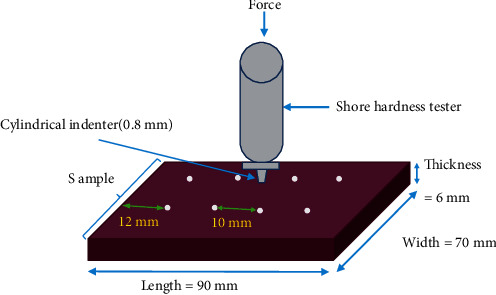
Schematic illustration of Shore A hardness indentations on a test sample (*n* = 12).

The Shore A hardness tester is a cylindrical indenter measuring 1.6 mm in diameter; it narrows to a blunt tip measuring 0.8 mm. It is allowed to stabilise for 10 minutes before the test. To minimise the effects of creep, the indenter is pressed on the specimen for one second of dwell time. Measurements were on a scale of 0 to 100 units, and each measurement was at least 10 mm apart from each other and 12 mm from the edge of the specimen [17] (Figure 2). Shore A hardness number was scored at 100 if no displacement occurred whereas a score of 0 was given to complete penetration. At predetermined time periods, measurements (*n* = 12 per sample per material) were taken: 1 hour after mixing, 24 hours after mixing, 72 hours after mixing, and 168 hours (1 week) after mixing [9, 18–20].

Shore A hardness values using the following were used to calculate Young's (elastic) moduli at all-time points for all commercial and experimental VPS samples: *H* = 100erf(*kE*^1/2^), where *H* is Shore A hardness value, *k* is 3.186 × 10^−4^ Pa^-1/2^, *E* is Young's modulus, and the erf is the error function (to generate a hardness scale).

## 3. Results

**Figure 3:**
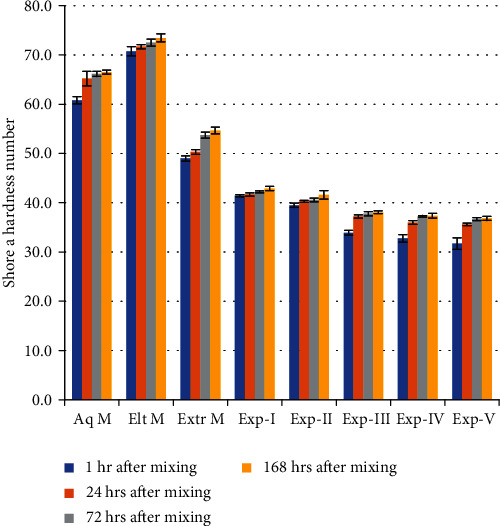
Shore A hardness of experimental and commercial VPS at four different time points (± standard errors; *n* = 12).

This study showed a significant increase (ANOVA, *p* < 0.05) in hardness at increasing time points (1 hour after mixing, 24 hours after mixing, 72 hours after mixing, and 168 hours after mixing) for all commercial and experimental VPS and can be appreciated in Figure 3.

All Exp formulations showed significantly lower values of Shore A hardness as compared to commercial VPS. At all-time points studied for commercial products, Elt M showed the highest values followed by Aq M then Extr M. Exp-I was found to be significantly harder than all other Exp VPS. The hardness of Exp-II decreased as compared to Exp-I (control), by the addition of TFDMSOS and was a further significant (*p* < 0.001) decrease after the addition of the surfactant into Exp-III, IV, and V. The Shore A hardness decreased for Exp-III, IV, and V at all-time points as the concentration of surfactant (2%, 2.5%, and 3%) increases. There was no significant difference between Exp-IV and V at 24 hours and Exp-III, IV, and V at 168 hours after mixing (Figure 3).

**Figure 4:**
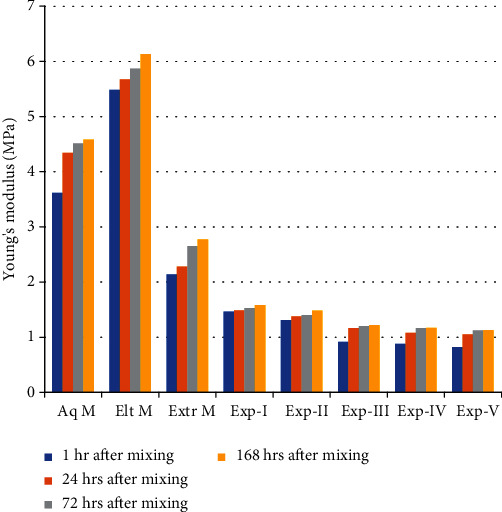
Young's modulus results of experimental and commercial VPS calculated from Shore A hardness results at four different time points.

It has been found that Shore A hardness and Young's moduli are directly proportional to each other as Young's moduli for all materials were calculated from Shore A hardness values (Figure 4).

## 4. Discussion

The novel experimental vinyl polysiloxane impression materials had comparatively lower hardness and Young's modulus yet these materials were within the normal range of stain-in-compression according to ISO4823 (2007) [15]. Therefore, the hypothesis of this study is accepted. As the storage time increases so does the hardness of all the materials studied as was demonstrated by the Shore A hardness values (Figure 3). The cross-linking process of polymerisation continues after the material is set [21]. The increase in hardness causes an increase in the Young's (elastic) modulus [2, 3].

The Shore A hardness for elastomeric impression materials was in the normal range, however for experimental VPS were lower than all commercial materials at all-time points [2, 3]. TFDMSOS in Exp-II decreased the hardness as compared to Exp-I control. The addition of surfactant (Rhodasurf CET-2) caused a further reduction in hardness. The reason for the decrease in hardness by the addition of TFDMSOS and Rhodasurf CET-2 is not clear.

Due to the molecular size of the novel cross-linking agent, the polymer chains are further apart forming a polymer matrix [22]. Increased cross-linking should improve other properties and contribute to a decrease in hardness while improving tear strength and % elongation-at-break [23]. This data shows that the lower the hardness of the experimental VPS the higher the strain in compression values, and this inversely correlates this data.

The hardness values for all materials at all-time points were directly correlated with Young's (elastic) modulus values. After being stored at room temperature (22°C) for 30 min, 60 min, 3 hours, 24 hours, 48 hours, 1 week, and 2 weeks period time, Papadogiannis et al. [9] investigated Young's modulus of elastomeric impression materials. Their results showed that increasing storage time increased Young's moduli for all materials. The polyether material was the stiffest (10.63-12.56 MPa). The Young's moduli ranged from 1.81 to 12.99 MPa; on comparing the results of the current study with Papadogiannis et al.'s results, it was seen that Impregum F had much higher Young's moduli compared to all commercial and Exp VPS investigated in the present study (0.82 to 6.13 MPa).

ISO4823 (2007) or ADA (1977) specification 19 do not give the normal range of hardness for elastomeric impression materials, and as far as Young's modulus is concerned, there are no set criteria. There is increased difficulty in removing the impression (and tray) from the mouth [2, 3, 24] when using stiffer impression materials with high Young's modulus.

## 5. Conclusion

- Commercial and experimental VPS impression materials exhibited higher Shore A hardness values with time (i.e.,1 hour after mixing, 24 hours after mixing, 72 hours after mixing, and 1 week after mixing); this was due to the continued polymerisation process after the materials had set
- Shore A hardness and Young's moduli were directly proportional to each other
- The hardness of the set commercial and experimental VPS materials was within the limits that set impression material can be removed from the mouth and the cast without permanent deformation
- Commercial and experimental materials had enough rigidity to contain the stone during pouring

## Figures and Tables

**Table 1:** Young's moduli of elastomeric impression materials calculated from Shore A hardness values [2, 7].

Materials	Brands	Shore A hardness	Young's modulus (MPa)		
			Calc.^∗^ direct method	Calc. error function (equation in text)	Calc. gent equation (equation in text)
VPS	Zerosil soft	54.5	3.5	3.2	2.9
	Zerosil super soft	53.1	3.0	3.0	2.8
	Zerosil light	56.7	3.5	3.4	3.2
	Zerosil mono	62.9	4.1	4.3	4.1
	Extrude	53.2	2.6	2.6	2.8
Polyether	Impregum PS	53.7	2.9	2.6	2.8

^∗^Calculations.

**Table 2:** Commercially available vinyl polysiloxane impression materials used in the study.

Commercial VPS	Lot/batch number	Manufacturers
Aquasil ultra monophase (Aq M)	090505	Dentsply, USA
Elite HD monophase (Elt M)	95503	Zhermack, Italy
Extrude (Extr M)	0-1068	Kerr, USA

## Data Availability

The datasets used and/or analysed during the current study are available from the corresponding authors on reasonable request.
